# Prognostic Value of Survivin in Nasopharyngeal Carcinoma: A Systematic Review and Meta-analysis

**DOI:** 10.7150/jca.46282

**Published:** 2021-05-27

**Authors:** Wenji Xie, Ouying Yan, Feng Liu, Yaqian Han, Hui Wang

**Affiliations:** Department of Radiotherapy, Hunan Cancer Hospital & The Affiliated Cancer Hospital of Xiangya School of Medicine, Central South University, 283 Tongzipo Road, Changsha 410013, Hunan, China.

**Keywords:** survivin, nasopharyngeal carcinoma, prognosis, meta-analysis

## Abstract

**Background:** Previous studies have shown that survivin has potential prognostic value in nasopharyngeal carcinoma. However, the results remained controversial until now. Thus, to investigate the influence of survivin expression on prognosis and clinical characteristics in nasopharyngeal carcinoma, we performed this meta-analysis.

**Methods:** We searched PubMed, PMC, Embase, Web of Science, Cochrane Library, and China National Knowledge Infrastructure electronic databases from their establishment to 1 March 2021. The pooled hazard ratio (HR) and the pooled odds ratio (OR) were used to evaluate the prognostic and clinicopathological values of survivin in nasopharyngeal carcinoma. We used the I^2^ statistic and the Q test to evaluate heterogeneity. Meta-regression, publication bias, and sensitivity analyses were also conducted.

**Results:** A total of 26 eligible studies with 2278 patients were included in our meta-analysis. We found that the expression of survivin is connected with poor overall survival (HR=1.94; 95% confidence interval (CI)=1.52-2.48; P<0.001), lymph node metastasis (OR=3.01; 95% CI=2.31- 3.91; P<0.001), local recurrence (OR=2.40; 95% CI=1.60-3.61, P<0.001), distant metastasis (OR=2.58; 95% CI=1.74-3.84, P<0.001), and a higher clinical stage (OR=4.58; 95% CI=2.81-7.47, P<0.001). However, no significant correlations were found between survivin expression and radio-sensitivity (OR=1.33; 95% CI=0.25-7.17, P=0.737) or gender (OR=1.02; 95% CI=0.75-1.39, P=0.887).

**Conclusions:** This meta-analysis indicates that survivin could be used as a biomarker for predicting prognosis in nasopharyngeal carcinoma.

## Introduction

Nasopharyngeal carcinoma (NPC) is a malignancy deriving from the nasopharynx epithelium. Because of its lopsided regional distribution and unique etiological risk factor, NPC has distinct characteristics compared with other head and neck cancer 1. The incidence of NPC in southeast Asia, norther Africa, and southern China is much higher than that in other areas of world 2. Moreover, more than 60% of newly diagnosed NPC patients are in China 3. Radiotherapy with or without chemotherapy is the mainstay treatment for NPC because of its high radio-sensitivity and special anatomical location 4, 5. Despite advances in radiotherapy technology and chemotherapy modality, survival outcomes of advanced NPC are still unsatisfactory 6. Locoregional recurrence and distant metastasis still remain the primary reasons for treatment failure, especially the later 7. To date, several prognostic indicators, like tumor-node-metastasis (TNM) stage 8, 9 and Epstein-Barr virus (EBV) DNA 10, have been used to evaluate the survival outcomes of NPC. However, those indicators cannot be accurate enough for predicting the prognosis because of biological heterogeneity and false positive rate 11, 12. Therefore, a novel and effective biomarker is urgently required to evaluate prognosis for NPC patients.

Survivin, also named baculoviral inhibitor of apoptosis repeat-containing 5 (BIRC5), is an important member of the inhibitors of apoptosis family 13. Survivin is highly expressed in embryonic tissues and tumors, but rarely expressed in normal adult tissues 14. It has been reported that survivin plays a key role in the inhibition of apoptosis, tumor cell proliferation, and tumor angiogenesis 15-17. A number of studies have demonstrated that survivin expression is associated with poor prognosis in various cancers, such as renal cell carcinoma 18, oral squamous cell carcinoma 19, and hepatocellular carcinoma 20.

However, the prognostic and clinicopathological value of survivin expression in patients with NPC remains ambiguity. Therefore, the aim of this meta-analysis is to investigate whether survivin is related to clinicopathological characteristics of NPC and evaluate its prognostic value.

## Materials and methods

### Search strategy

This study was conducted according to the Preferred Reporting Items for Systematic Reviews and Meta-Analyses (PRISMA) criteria 21.

A systematical literature retrieval was conducted without language restriction. We searched PubMed, PMC, Web of Science, Embase, Cochrane Library, and China National Knowledge Infrastructure (CNKI) databases by using the following syntax: (“survivin” or “baculoviral inhibitor of apoptosis repeat-containing 5” or “BIRC5”) and (“NPC” or “nasopharyngeal carcinoma” or “nasopharyngeal cancer”). The final search was conducted on Mar 1, 2021. Also, the reference lists of relevant literatures were searched manually for potential eligible studies.

### Inclusion and exclusion criteria

Eligible studies must meet the following inclusion criteria: (a) NPC patients were confirmed by histopathological diagnosis and without other malignances; (b) studies focused on the association between survivin expression and survival outcomes or clinical variables of NPC patients; (c) survivin expression was detected by immunohistochemistry (IHC); (d) sufficient data was provided to extract or estimate hazard ratio (HR) and odds ratio (OR) with 95% confidence intervals (CIs). The exclusion criteria were as follows: (a) reviews, meta-analysis, letters, comments, case reports and conference abstracts; (b) the study based on cells or animal models; (c) the study failed to provide sufficient data for acquiring HR, OR and 95% CI. Two independent investigators assessed the selected studies.

### Data extraction and quality assessment

Data was extracted independently by two investigators. The following required data was retrieved from each included study: last name of first author, year of publication, sample size of the study, country of patients, follow-up duration, data of clinical characteristics (age, gender, stage, radio-sensitivity, lymph node metastasis, distant metastasis, and local recurrence), HR and its 95% CI for survival, Kaplan-Meier curves for survival, definition of survivin expression, and positive rate of survivin. If the HRs with 95% CIs were not directly reported, we calculated them from Kaplan-Meier curves 22. Each included study's quality was assessed by the Newcastle-Ottawa Scale (NOS). The study with ≥6 points on NOS was evaluated as high quality.

### Statistics analysis

Pooled estimates of OR and HR with 95% CI were applied to evaluate the relationship of survivin expression with NPC clinical characteristics and prognosis, respectively. Q test and I^2^ statistic were used to conduct the heterogeneity test 23. When I^2^ value >50% or P value <0.1, which suggested significant heterogeneity, a random-effect model would be applied to calculate the estimate. Otherwise, a fixed-effect model would be used. Meta-regression was performed to evaluate the effects of the definition of survivin positivity and the level of survivin expression on the HR for OS. Meanwhile, Begg's test and Egger's test were used to calculate publication bias 24, 25. In addition, the stability of the results was assessed by the sensitivity analysis 26. All statistical analyses were performed by STATA 12.0 (STATA Corporation, College Station, TX, USA).

## Results

### Literature characteristics and selection

**Figure 1:**
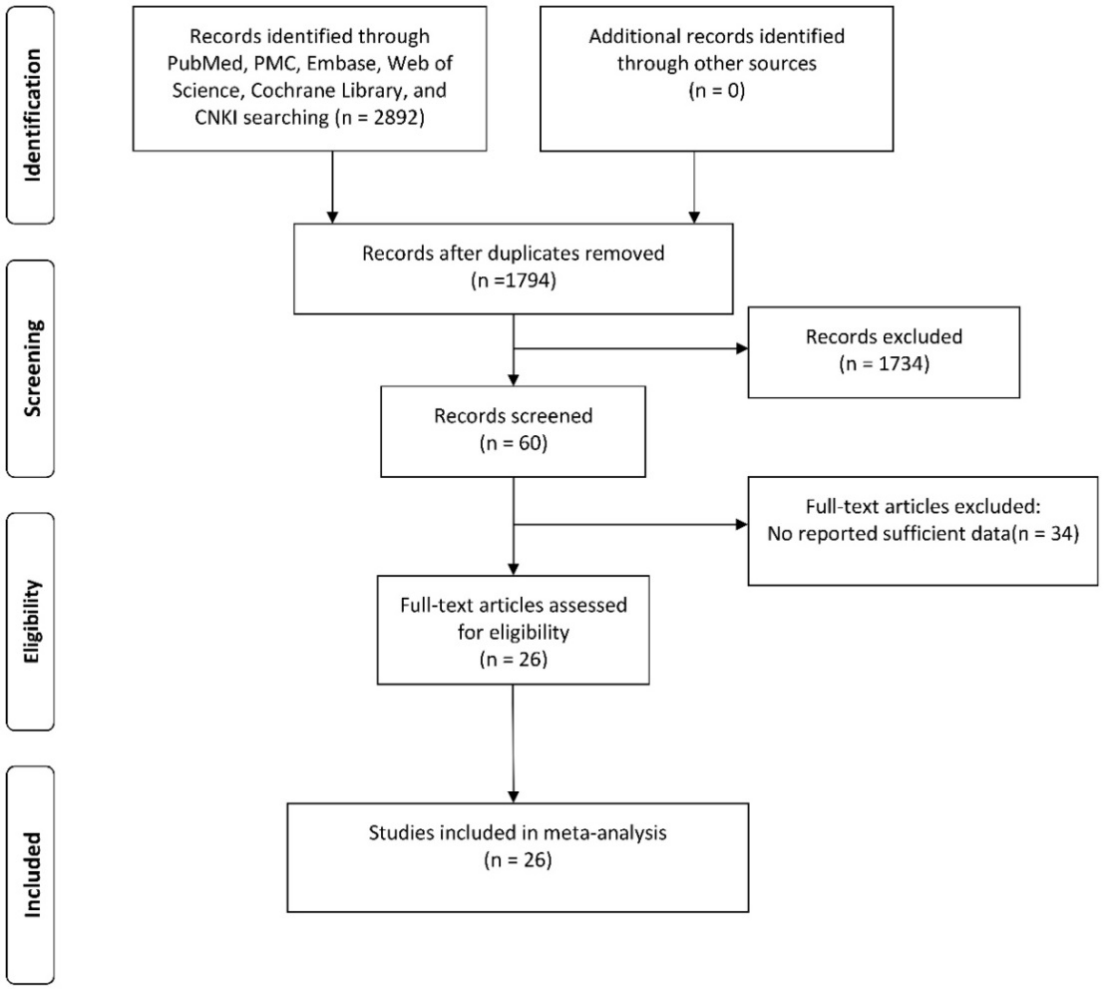
Flowchart presenting the steps of literature search and selection.

As shown in Figure 1, a total of 1794 potential studies were retrieved after duplicates were discarded. After reading the titles and abstracts, 1734 articles were excluded because they were conference abstracts, reviews, not human studies, case reports, or unrelated studies. After reading 60 full-text articles, a further 34 studies were excluded: 8 studies did not use immunohistochemical method for detection, 24 studies lacked sufficient data, and 2 studies showed overlapping data.

Consequently, a total of 26 studies 27-52 with 2278 patients were included in this meta-analysis. The main characteristics of the 26 eligible studies are summarized in Table 1. All the contained studies were designed retrospectively and were published between 2004 and 2019. Only one article was conducted in Canada, and the rest were performed in China. There were 8 studies presenting the relationship between survivin expression and overall survival (OS) in NPC patients. The NOS was applied to assess the quality assessment of all eligible studies, and the mean score of included studies was 6.6 (range 6-8).

### Survivin and survival

**Figure 2:**
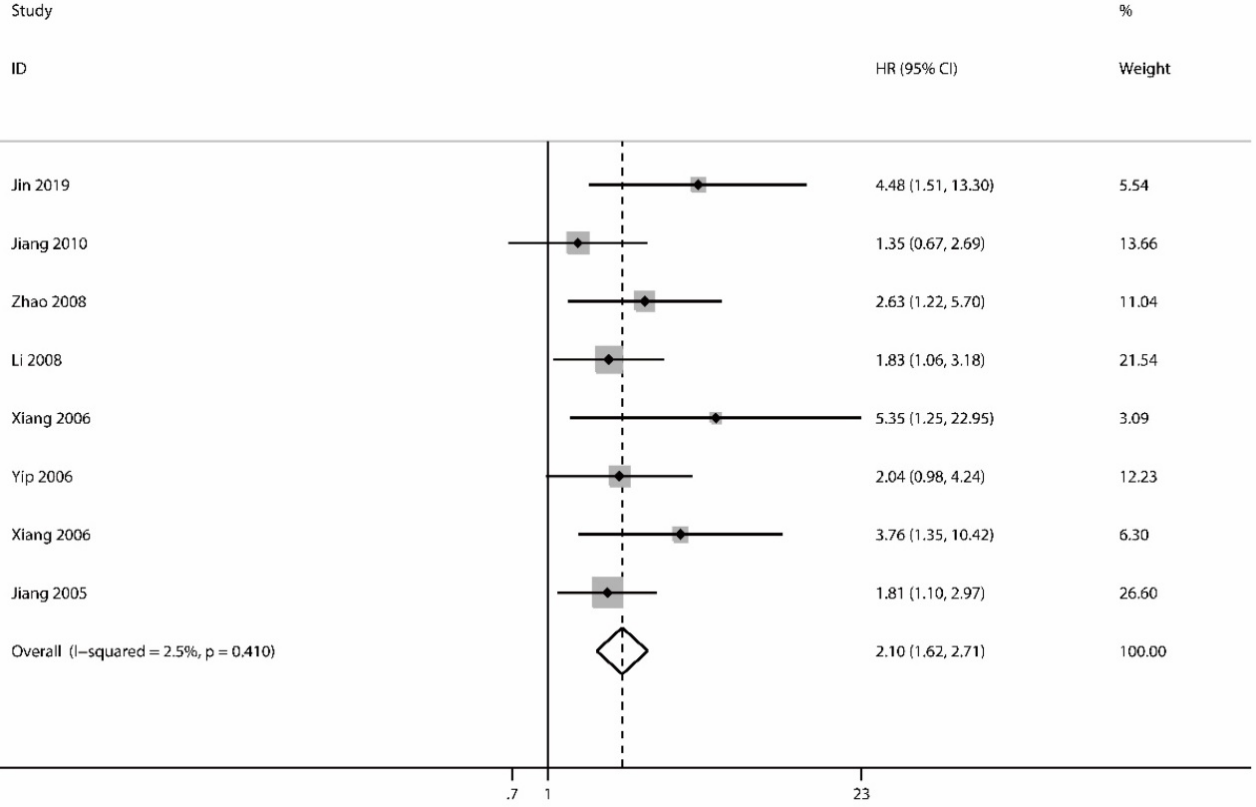
Forest plot depiction of survivin expression and hazard ratio (HR) for overall survival.

The analysis of OS included 8 studies 32, 37, 43, 44, 47-50 with 910 patients. The heterogeneity test showed no significance (I^2^=2.5%; P=0.410); however, we found a significant association between survivin and OS. The expression of survivin was associated with a poor OS for NPC patients in a fixed-effects model (HR=2.10; 95% CI=1.62-2.71; P<0.001) (Figure 2).

### Survivin and clinical characteristics

**Figure 3:**
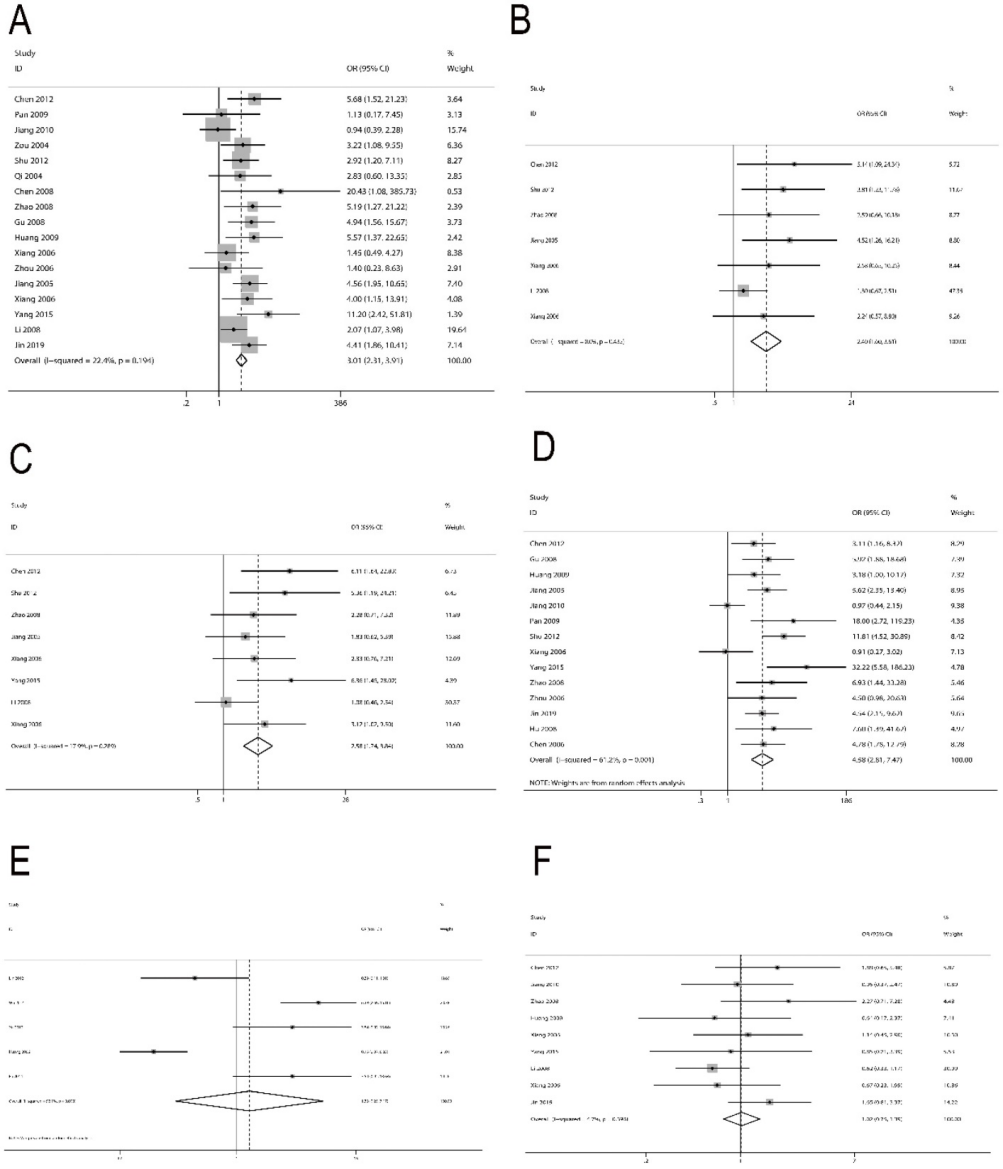
Forest plot depiction of survivin expression and odds ratio (OR) for clinical characteristics. **A.** Lymph node metastasis vs no lymph node metastasis. **B.** Local recurrence vs no local recurrence. **C.** Distant metastasis vs no distant metastasis. **D.** III/IV vs I/II. **E.** Radio-sensitivity vs radio-resistance. **F.** Male vs female.

We also analyzed the relationship between survivin and NPC clinical features. For lymph node metastasis, 17 studies 30-38, 40-45, 47, 50 with 1450 patients were included. The result indicated a significant correlation between survivin expression and lymph node metastasis (OR=3.01; 95% CI=2.31-3.91; P<0.001) (Figure 3A). For local recurrence, 7 studies 30, 34, 37, 43, 44, 47, 48 with 769 patients were included. We detected that survivin expression was associated with local recurrence (OR=2.40; 95% CI=1.60-3.61, P<0.001) (Figure 3B). For distant metastasis, 8 studies 30, 34, 37, 43-45, 47, 48 with 814 patients were included. The result also presented an obvious relevance between survivin expression and distant metastasis (OR=2.58; 95% CI=1.74-3.84, P<0.001) (Figure 3C). For clinical stage, 14 studies 30-32, 34, 37, 38, 40-43, 45, 50-52 with 1089 patients were included. Survivin expression was found to be prominently associated with more advanced clinical stage (OR=4.58; 95% CI=2.81-7.47, P<0.001) (Figure 3D). However, we did not observe a significant correlation between survivin expression and radio-sensitivity (OR=1.33; 95% CI=0.25-7.17, P=0.737) (Figure 3E) or gender (OR=1.02; 95% CI=0.75-1.39, P=0.887) (Figure 3F).

### Sensitivity analysis, meta-regression analysis and publication bias

**Figure 4:**
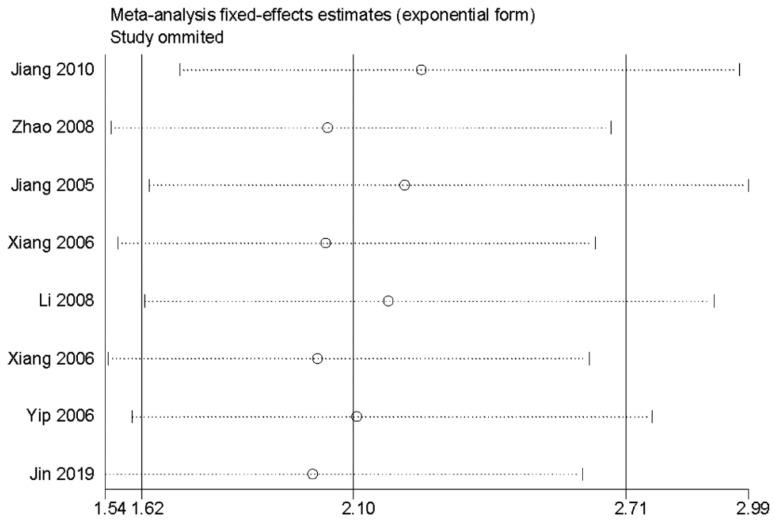
Sensitivity analysis for the association between survivin and overall survival.

**Figure 5:**
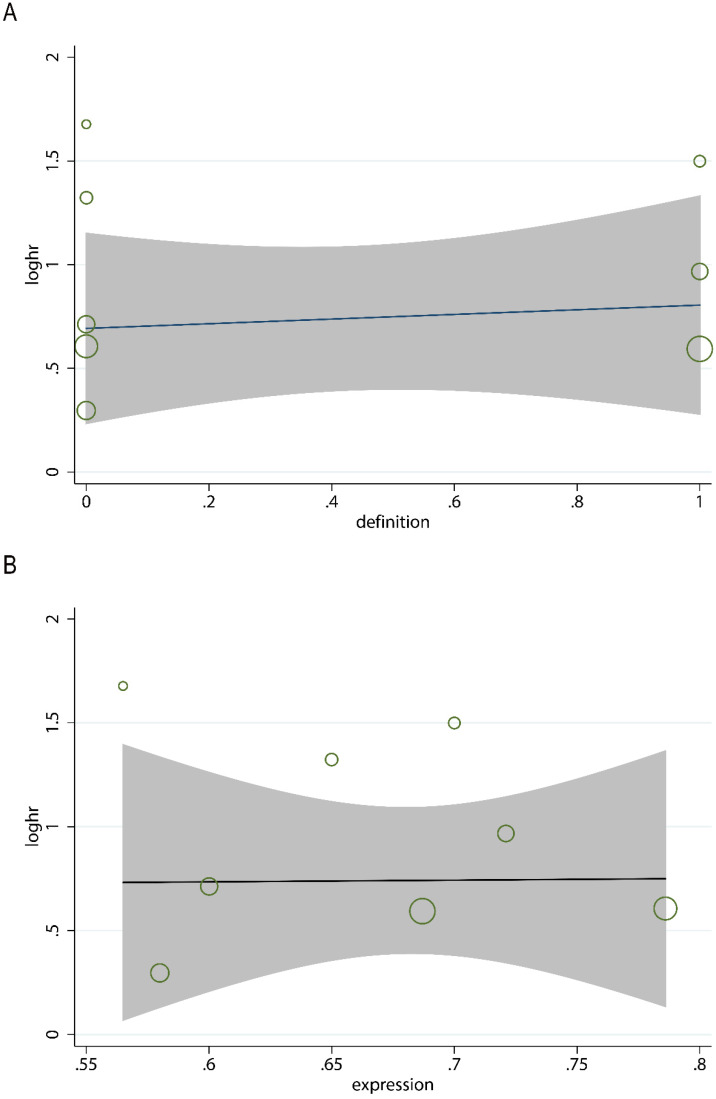
Meta-regression analysis for the association between the definition of survivin positivity (**A**), the level of survivin expression (**B**), and overall survival.

**Figure 6:**
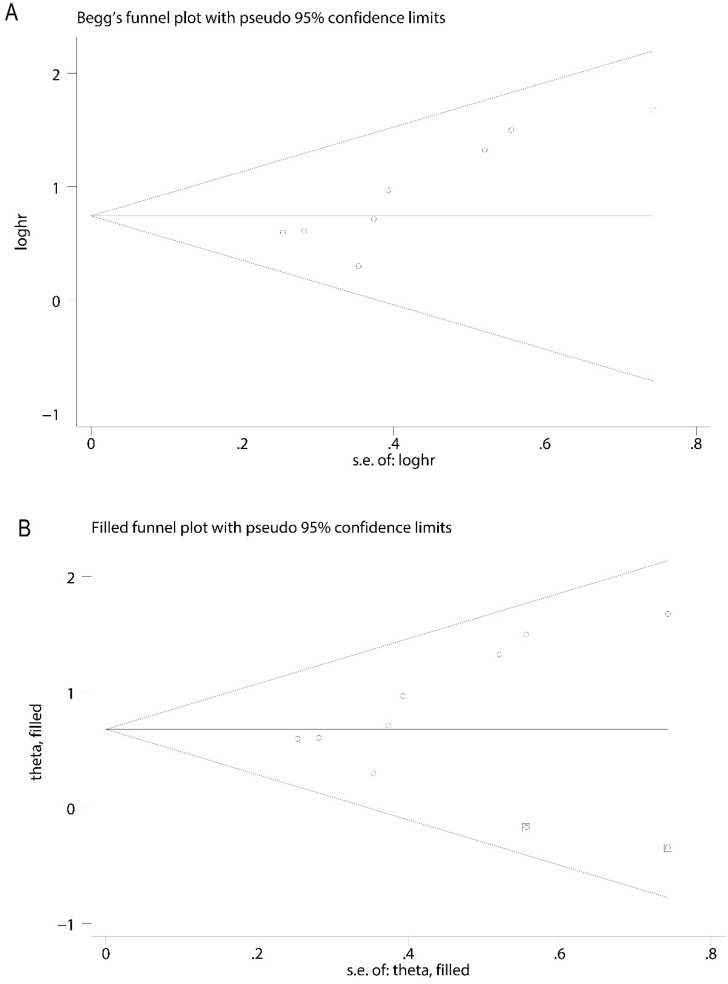
Funnel plot for publication bias analysis. **A.** The asymmetry of funnel plot was found, indicating possible publication bias. **B.** The trim and fill method was performed to rectify analysis, and no visible asymmetry was found in the funnel plot.

In order to evaluate the variations, we conducted sensitivity analyses of OS. There were no obvious variations in the results, which shows their stability (Figure 4). Moreover, in meta-regression analysis, we did not identify the definition of survivin positivity (P=0.707) or the level of survivin expression (P=0.969) that was effect modifiers for influence of survivin on OS (Figure 5). Publication bias was detected in OS (Begg's test Pr>|z|=0.006, Egger's test P>|t|=0.011). Accordingly, we used a trim and fill method to estimate the asymmetry in the funnel plot. The result had not materially altered (HR=1.94; 95% CI=1.52-2.48; P<0.001) (Figure 6).

## Discussion

In the present meta-analysis, the pooled data showed promising prognostic value of survivin for NPC patients. We detected that survivin expression was related to poor OS in NPC. The risk of death in patients with survivin expression was 1.94 times higher than patients without survivin expression. Furthermore, we also explored the relationship between survivin and clinical characteristics and found that survivin was related to a higher clinical stage, positive lymph node, local recurrence, and distant metastasis. Taken together, detection of survivin in NPC patients could help evaluate the malignant degree and predict prognosis.

Our research was consistent with many previous studies exploring the prognostic role of survivin in other cancers. A meta-analysis of 11 studies demonstrated that survivin expression was significantly associated with poor OS (HR=2.28; 95% CI=1.57-3.33; P<0.001) and poor cancer-specific survival (HR=2.08; 95% CI=1.07-4.05; P=0.032) in renal cell carcinoma 53. Another meta-analysis also showed that higher survivin expression could predict worse OS (HR=1.96; 95% CI=1.57-2.45; P<0.001) in patients with glioma 54. In non-small cell lung cancer, survivin expression was related to lymph node metastasis, TNM stage, and histological differentiation 55. All these previous researches had indicated that survivin might serve as an important prognostic biomarker for cancer patients. In this meta-analysis, we showed the positive association of survivin with survival and clinical characteristics in NPC. However, we failed to detect any relationship between survivin and radio-sensitivity. This may due to the limited number of radio-sensitivity studies included.

The possible reason why survivin presents the prognostic value in NPC patients might be that survivin plays a key role in cell apoptosis. Survivin is composed of 142 amino acids and has a strong effect of inhibiting apoptosis 56. Caspases play an important role in the process of apoptosis 57. It has been shown that survivin blocks the process of apoptosis through directly inhibiting caspase activation 58. The anti-apoptosis effect is closely related to the treatment resistance. It has been reported that anti-survivin siRNA enhances apoptosis and overcomes chemotherapy resistance *in vitro* 59 and *in vivo* 60. In NPC, Shi et al. reported that the YM-155, which is a survivin inhibitor, induces apoptosis of NPC cells and inhibits tumor growth in the mouse model 61. Apart from the inhibition of apoptosis, cell proliferation is also related to survivin. During the G2/M phase of the cell cycle, survivin is expressed and promotes cell division by binding to spindle tubulin 62, 63. Furthermore, it has also been reported that survivin plays an important role in tumor angiogenesis 64. Taken together, it is believed that survivin could be an appropriate biomarker and a potential therapeutic target for prognosis prediction and precision therapy.

There were several limitations in this meta-analysis. First, a publication bias was detected in OS. This may due to the limited number of included studies (n=8). The pooled data changed slighted but remained significant after the trim and fill method was conducted. Second, only English and Chinese literatures were included, selection bias and recall bias may exist in this meta-analysis. Third, because some studies did not directly report HRs, we extracted HRs from Kaplan-Meier curves. This may have influenced the accuracy of the results. Fourth, most of the included studies were published at 5 to 15 years ago, the last study was published in 2019. Taking into account these limitations, these results must be interpreted with caution when used in current clinical practice, and more studies are needed to verify our findings.

In conclusion, our meta-analysis demonstrated the prognostic value of survivin in patients with NPC. The expression of survivin can predict poor prognosis of NPC. Therefore, survivin might be served as a promising biomarker in survival prediction and targeted therapy. In the future, larger well-designed prospective studies are needed to verify our findings.

## Figures and Tables

**Table 1:** The main characteristics of included studies

First author	Year	Country	No. of patients	Median/Mean age (range), years	Antibody source	Definition of positivity	Cut-off value	Survivin + (%)	Study design	NOS
Lin 27	2012	China	72	47.8 (14-78)	MXB	SS	Sum≥3	73.6	R	6
Wu 28	2014	China	106	NM	NM	Per	51%	NM	R	7
Xu 29	2012	China	72	41 (20-69)	MXB	Per	26%	72.2	R	6
Chen 30	2012	China	80	48 (26-72)	Santa Cruz	SS	Pro≥3	61.3	R	8
Pan 31	2009	China	32	46 (16-64)	ZSGB BIO	Per	6%	68.7	R	6
Jiang 32	2010	China	105	43 (15-72)	MXB	Per	6%	58.0	R	7
Zou 33	2004	China	96	48 (18-73)	MXB	Per	6%	75.0	R	6
Shu 34	2012	China	128	47.2 (18-67)	NM	SS	Pro≥1	70.3	R	6
Qi 35	2004	China	33	46 (28-69)	Santa Cruz	SS	Pro≥1	66.7	R	7
Chen 36	2008	China	50	45	ZSGB BIO	Per	10%	88.0	R	6
Zhao 37	2008	China	68	53 (22-74)	MXB	SS	Sum≥2	72.1	R	6
Gu 38	2008	China	68	49.8	MXB	Per	5%	72.1	R	6
Huang 39	2006	China	230	44.6 (7-83)	Santa Cruz	SS	Pro≥3	83.5	R	6
Huang 40	2009	China	57	51 (21-78)	ZSGB BIO	SS	Pro≥3	68.4	R	7
Xiang 41	2006	China	68	45.6	MXB	Per	10%	69.1	R	6
Zhou 42	2006	China	43	54.9 (22-74)	MXB	SS	Sum≥2	76.7	R	7
Jiang 43	2005	China	115	45.8 (18-65)	MXB	SS	Pro≥1	68.7	R	6
Xiang 44	2006	China	92	45 (18-71)	Santa Cruz	Per	5%	56.5	R	7
Yang 45	2015	China	45	(26-71)	NM	Per	6%	71.1	R	7
Fu 46	2014	China	72	41 (20-69)	MXB	Per	26%	72.2	R	6
Li 47	2008	China	206	NM	Santa Cruz	Per	5%	78.6	R	7
Xiang 48	2006	China	80	NM	Santa Cruz	Per	5%	65.0	R	8
Yip 49	2006	Canada	80	NM	Novocastra Lab	Per	5%	60.0	R	6
Jin 50	2019	China	164	45 (24-70)	Santa Cruz	SS	Sum≥3	70.0	R	8
Chen 51	2006	China	83	NM	Santa Cruz	Per	5%	66.2	R	6
Hu 52	2008	China	33	46 (28-69)	Santa Cruz	SS	Pro≥1	66.7	R	7

No., number; NM, not mentioned; SS, score system combining intensity and percentage; R, retrospective; Per, percentage of positive cells; Sum, the sum of intensity score and percentage score; Pro, the product of intensity score and percentage score; NOS, Newcastle-Ottawa Quality Assessment Scale.
